# MENDing Recovery: Comprehensive Perioperative Care Cuts Hospital Stay After Minimally Invasive CABG

**DOI:** 10.1177/15569845251361492

**Published:** 2025-07-31

**Authors:** Christine Ashenhurst, Omar Toubar, Menaka Ponnambalam, Roy Masters, Ming Hao Guo, Hugo Issa, Marc Ruel

**Affiliations:** 1 Division of Cardiac Surgery, University of Ottawa Heart Institute, ON, Canada; 2 Faculty of Medicine, University of British Columbia, Vancouver, BC, Canada; 3 Faculty of Medicine, McGill University, Montreal, QC, Canada

**Keywords:** minimally invasive, coronary artery bypass grafting, comprehensive care, perioperative optimization, length of stay

## Abstract

**Objective:**

To evaluate the impact of a novel multidisciplinary initiative, known as the Multimodal ENhanced Discharge (MEND), on length of stay (LOS) for patients undergoing minimally invasive coronary artery bypass grafting (MICS CABG).

**Methods:**

The MEND program aims to optimize the patient’s preoperative condition and increase preparedness, provide individualized perioperative care, and ensure early postdischarge follow-up to support active recovery and facilitate early discharge. This single-center, retrospective analysis reviewed LOS and readmission data for 198 consecutive patients who underwent MICS CABG by a single surgeon. Of these, 91 patients received routine care (RC) and 107 patients received care through the MEND program.

**Results:**

The median ward (non–intensive care unit) LOS was significantly shorter by 33% in the MEND group versus the RC group (2 vs 3 days, *P* < 0.001), resulting in a 40% shorter median total hospital LOS in the MEND group versus the RC group (2 vs 5 days, *P* < 0.001). Readmission rates were 14.3% for RC and 6.6% in the MEND group (*P* = 0.12).

**Conclusions:**

Implementation of the MEND program in patients undergoing MICS CABG was associated with significantly shorter overall hospital LOS without an increase in readmission rates. No statistically significant differences in baseline characteristics between the RC and MEND cohorts were observed. These findings suggest MEND is an effective and generalizable program for optimizing recovery. Ultimately, this model of care has the potential to positively affect health care costs, improve surgical wait times, and expand capacity in MICS CABG programs.

## Graphical abstract

**Visual abstract:**
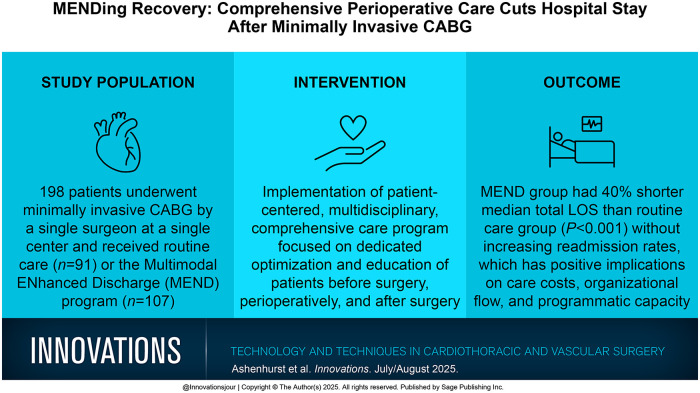

**Central Message**

We evaluated hospital LOS in 198 MICS CABG patients at a single center before and after implementation of the Multimodal ENhanced Discharge program, a patient-centered, multidisciplinary, comprehensive perioperative care plan. There was a 40% reduction in median total LOS after implementing the program, without increasing readmission rates.

## Introduction

For patients with coronary artery disease (CAD), coronary artery bypass grafting (CABG) provides both symptomatic relief and prognostic benefits.^1–3^ The advent of a minimally invasive cardiac surgery approach for CABG (MICS CABG), undertaken via left minithoracotomy and usually performed without the use of cardiopulmonary bypass (CPB), has been a landmark development in surgical revascularization.^4^ Although MICS CABG involves a substantial learning curve for the surgeon, studies have shown outcomes comparable to traditional sternotomy-based CABG.^5–10^ Observational data indicate that the less invasive option reduces recovery time, which is desirable for patients as well as for programs.^10,11^

**Fig. 1.**
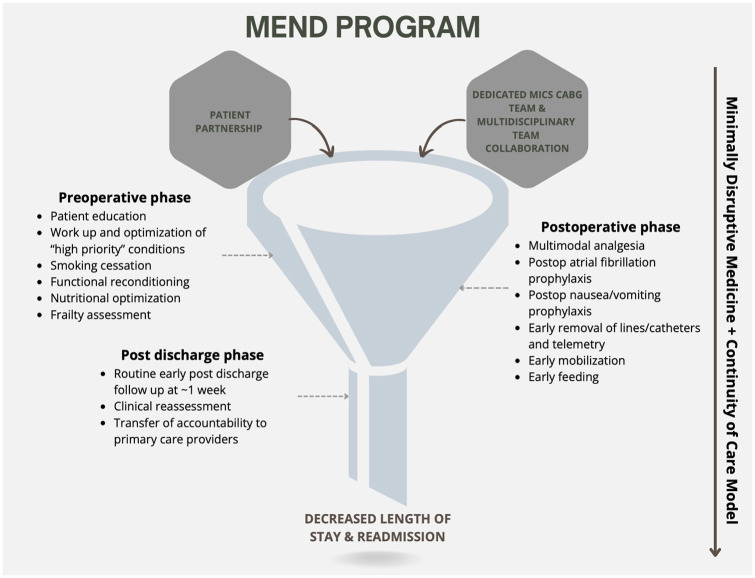
A funnel diagram depicting the key components of the MEND program. MEND, Multimodal ENhanced Discharge.

**Fig. 2.**
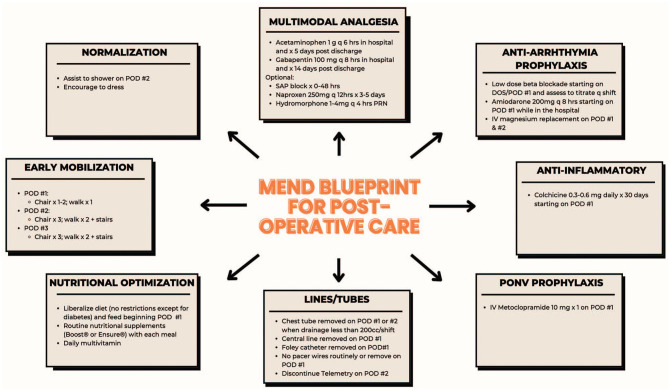
MEND blueprint for postoperative care. DOS, day of surgery; MEND, Multimodal ENhanced Discharge; POD, postoperative day; PONV, postoperative nausea and vomiting; SAP, serratus anterior plane.

The avoidance of a sternotomy for surgical revascularization creates an opportunity for matched modernization of the perioperative care of patients undergoing MICS CABG. In response to a programmatic goal of reducing length of stay (LOS) after MICS CABG, the “Multimodal ENhanced Discharge” (MEND) program was developed. Recovery after surgery is a multidimensional process with physiologic, functional, psychological, and social factors.^12,13^ Although guideline-based strategies that enhance recovery have long been embedded in our institution’s intraoperative and intensive care unit (ICU) protocols, the MEND program was developed to address the preoperative, postoperative (non–ICU), and postdischarge phases of care. MEND integrates Enhanced Recovery After Surgery (ERAS) principles with the Continuity of Care Model (CCM) and Minimally Disruptive Medicine (MDM).^12–15^ A central feature of MEND is the dedicated MICS CABG team, composed of 1 nurse practitioner and 1 surgeon, working collaboratively with the multidisciplinary team to provide care through all phases of the patient journey. In keeping with the CCM, this structure is intentionally designed to minimize fragmentation in care as a means of reducing LOS and readmissions.^14^ Another defining element is the MEND program’s early initiation-beginning at the time of surgical referral-using this period to educate, assess, and optimize patients prior to their surgical consultation. Subsequent efforts through MEND focus on improving patient preparedness, including managing expectations and discharge planning prior to surgery, followed by individualized postoperative care, and in-person early postdischarge follow-up. The schematics of evidence-informed care provided by MEND are illustrated in Figure 1 and Figure 2.

Although much of the current cardiac surgery research is focused on technical advancement, this study aims to highlight the impact of a dedicated panoramic perioperative approach on LOS in patients undergoing MICS CABG.

## Methods

### Patients

In this single-center, retrospective analysis, 198 patients underwent MICS CABG by a single surgeon from January 2018 to December 2023. Among them, 91 underwent surgery from January 2018 to December 2020 and are referred to as the routine care (RC) group. The institution introduced the MEND program in January 2021, and 107 patients who underwent MICS CABG from January 2021 to December 2023 are identified as the MEND group. LOS and readmission data were extracted from the hospital’s electronic medical record (EMR).

### Intervention

#### Preoperative phase

Drawing from the MDM model, the MEND program leverages the time between the surgical referral and surgery to foster patient partnership and strengthen health literacy. This includes education on CAD and coexisting conditions, the indication and conduct of surgery, timelines and expectations of recovery, and anticipating early discharge.

Early clinical assessments during this time allow for the workup and medical optimization of several high-priority conditions, including, but not limited to, iron-deficiency anemia, diabetes, smoking, chronic obstructive pulmonary disease (COPD), obstructive sleep apnea (OSA), decreased left ventricular function, pulmonary hypertension, and frailty.

Patients with preoperative anemia (defined as hemoglobin levels of less than 12 g/dL in females and 13 g/dL in males) and iron deficiency receive iron supplementation (both oral and intravenous administration) for anemia optimization.^16–18^ The target glycemic control for patients with diabetes undergoing CABG is a hemoglobin A1C of <7%.^14^ Together with community partners, patients with levels above target receive not only intensification of medical management but also counseling to ensure a clear understanding of their role in achieving glycemic control as a key contributor to optimal surgical outcomes.^14,15,18,19^ Smoking status is routinely identified at the time of referral for surgery, and all patients who smoke are advised to quit.^19^ Referrals to formal smoking cessation programs are placed, noting that layered efforts that use counseling, print and digital resources, nicotine replacement therapy, and pharmacotherapy (with bupropion and varenicline) result in the most success.^20,21^ Guideline-directed medical therapy is carefully implemented for patients with decreased left ventricular function and pulmonary hypertension, in collaboration with primary care providers and cardiologists both before and after surgery.^3,22^ Patients with actual or suspected COPD and OSA undergo formal assessments, with or without referrals to respirologists when applicable. Patients >65 years of age are objectively screened for frailty using the Essential Frailty Toolset.^23^ Patients are generally advised to increase functional capacity through exercise, and those formally assessed to be frail are advised to engage in a beginner walking program and referred to a physiotherapist for reconditioning.^14,15,19^ Patients are encouraged to increase protein intake pending surgery, and for patients with a serum albumin level less than 3.5 g/dL, print resources to guide increasing protein intake are provided, and a referral to the dietitian for formal nutritional optimization is placed.^14,23^ Regardless of the chronological age of the patient, when frailty and cognitive dysfunction were noted, their association with postoperative delirium is reviewed with patients and their families to better anticipate postdischarge needs and is also raised with the anesthesia team for mitigation strategies in the intraoperative and postoperative periods.^24^

#### Postoperative phase

Management of conditions requiring optimization in the preoperative period (Fig. 1) was continued through the postoperative period. The MEND “blueprint” for postoperative care, using evidence-informed practice, is outlined in Figure 2. In contrast to protocolization, MEND supports individualized application of care within the framework of the blueprint. In keeping with the CCM, the involvement of a consistent MICS CABG team through all phases of care was essential in reducing the fragmentation.

MICS CABG performed through a thoracotomy preserves the integrity of the bony thorax, thereby permitting unrestricted mobilization after surgery. The thoracotomy requires manipulation of the ribs, pleura, intercostal nerves, costovertebral joints, and muscles; unmanaged pain may adversely affect recovery and longitudinal quality of life scores.^25^ Drawing from the literature, MEND patients receive around-the-clock multimodal analgesia with acetaminophen, nonsteroidal anti-inflammatory drugs, and gabapentin (Fig. 2), respecting typical caveats for these drugs.^26–28^ Narcotic analgesia and serratus anterior plane blocks are used on an as-needed basis.

Postoperative atrial fibrillation (POAF) after cardiac surgery is estimated to have an incidence of 15% to 40% and is associated with stroke and increased LOS.^3,15^ MEND patients receive aggressive prophylaxis for POAF after surgery with beta-blockers, amiodarone, and intravenous magnesium in accordance with evidence-informed care.^15,29^ Noting emerging evidence linking the retention of pericardial blood with POAF, a posterior pericardiotomy was routinely undertaken at the time of surgery.^29^

MEND supports an active “demedicalization” of the patient after surgery with a view of reestablishing normalcy, thereby facilitating rapid discharge. Invasive vascular access and urinary catheters are routinely discontinued on the first postoperative day, and telemetry is removed at 48 h, when appropriate. Early mobilization pathways encourage patients to walk beginning on the first postoperative day. Patients are assisted to shower and dress on day 2 to encourage active “normalization” after surgery and confer confidence for an early discharge. Early resumption of nonrestricted diets is promoted by the MEND program, and aggressive treatment and routine prophylaxis of postoperative nausea and vomiting are implemented to ensure tolerance of this.^14,15^

#### Postdischarge phase

The absence of readmissions after hospitalization is a key indicator of a safe and satisfactory discharge. Conditions requiring optimization in the preoperative period and management in the postoperative period continue to be followed in the postdischarge phase. An in-person assessment with the MICS CABG team occurring approximately 1 week after discharge is a crucial component of the MEND program. This follow-up alleviates patient anxieties about transitioning from the hospital to home.^30,31^ In addition, it enables timely clinical reassessments and interventions, allows for medication reviews to ensure targeted management—particularly for secondary prevention after CABG—and safeguards transfer of accountability to community providers for longitudinal care.^31^

### Data Collection and Outcomes Measured

Key patient demographics and comorbidities (age, sex, body mass index, presence of left ventricular dysfunction, presence of type 2 diabetes mellitus, creatinine on the date of surgery, and smoking status at time of consultation) and time in days from the cardiac catheterization (and presumed referral) to surgery, as well as procedural characteristics, LOS, and readmissions were obtained from an EMR review.

The primary outcome was the total LOS in the hospital, which was divided into time spent in the ICU and on the ward (non-ICU). Secondary outcomes included wait time, defined as time from cardiac catheterization to date of surgery, and readmissions, defined as emergency or unplanned presentations to the hospital with complications associated with surgery, including atrial fibrillation, myocardial infarction, pleural/pericardial effusion, surgical site infection or dehiscence, or pericarditis. It was only possible to capture internal readmissions in this analysis.

Categorical variables are reported as counts and percentages and were compared using the chi-square test or Fisher’s exact test where appropriate. Continuous variables are reported as mean ± standard deviation or median with interquartile range (IQR) and compared with Student’s *t* test or Wilcoxon rank-sum test where appropriate. A *P* value of <0.05 was considered statistically significant. Statistical analyses were performed using R Studio version 2023.12.0+369 (Posit, Boston, MA, USA).

### Ethics Approval

All procedures performed in studies involving human participants were in accordance with the ethical standards of the institutional and/or national research committee and with the 1964 Helsinki Declaration and its later amendments or comparable ethical standards. The University of Ottawa Heart Institute Research Ethics Board approved this retrospective, observational research and waived individual research consent (ID: 20220626-01H).

## Results

A comparative analysis of key patient characteristics and comorbidities, wait times, and surgical details is shown in Table 1. In the RC cohort, the median age was 66 years (IQR 59.5 to 73.0 years), and 77% were male patients. In the MEND cohort, the median age was 65 years (IQR 59.0 to 71.5 years), and 82% were male patients. There were no statistically significant differences in age, sex, body mass index, creatinine on the day of surgery, presence of left ventricular dysfunction (defined as left ventricular ejection fraction <50%), concurrent diabetes, or active smoking status between the cohorts.

**Table 1.** Preoperative Characteristics of Routine Care and MEND Patients.

	Routine care *n* = 91	MEND *n* = 107	*P* value
Sex			0.45
Female	21 (23)	19 (18)	
Male	70 (77)	88 (82)	
Age, years	66 (59.5–73.0)	65 (59.0–71.5)	0.36
BMI, kg/m^2^	27.4 (25.0–30.7)	26.8 (24.1–30.2)	0.28
Creatinine on DOS, mg/dL	78.0 (67.5–90.0)	76.0 (65.0–86.0)	0.27
LV dysfunction^a^	13 (14)	13 (12)	0.82
Type 2 diabetes mellitus	19 (21)	31 (29)	0.25
Active smoker at time of consult	14 (15)	15 (14)	0.94
Status			0.52
Urgent	32 (35)	32 (30)	
Elective	59 (65)	75 (70)	
Out-of-town elective	8 (8.8)	10 (9.3)	
Wait time, days			
All patients	45.0 (13.0–93.5)	42.5 (10.0–85.0)	0.69
Urgent only	9.0 (6.0–15.3)	6.0 (4.5–8.5)	0.057
Elective only	68.0 (43.5–116.5)	66.0 (40.0–99.5)	0.61
Out-of-town elective	142 (141.5–215)	99 (65.8–180.8)	0.35

Abbreviations: BMI, body mass index; DOS, day of surgery; LVEF, left ventricular ejection fraction; MEND, Multimodal ENhanced Discharge.

Data are reported as median (interquartile range) or *n* (%).

a LV dysfunction defined as LVEF <50%.

There were no statistically significant differences in the number of urgent or elective cases or wait times between the 2 cohorts (Table 1). Patients traveling from out of town for elective surgery represented 8.8% of all RC cases (*n* = 8) and 9.3% of all MEND cases (*n* = 10). In most cases, out-of-town patients had received surgical consultations for sternotomy CABG in their home jurisdictions before being referred for minimally invasive CABG. Surgical planning was often influenced by logistical considerations related to travel for care, including patient preference.

Surgical details are presented in Table 2. More than 60% of RC cases (*n* = 55) were single-vessel CABG, and 39.6% were multivessel CABG (*n* = 36). Of MEND cases, 50.4% (*n* = 54) were single-vessel CABG and 49.5% (*n* = 53) were multivessel CABG (*P* = 0.21). CPB was used in 7 cases (7.7%) and 6 cases (5.6%) in the MEND group (*P* = 0.76). There were no statistically significant differences in the number of grafts or use of CPB between the 2 cohorts.

**Table 2.** Surgical Details of Routine Care and MEND Patients.

	Routine care *n* = 91	MEND *n* = 107	*P* value
Revascularization			0.21
SVST	55 (60.4)	54 (50.4)	
MVST	36 (39.6)	53 (49.5)	
CPB use	7 (7.7)	6 (6.6)	0.76

Abbreviations: CPB, cardiopulmonary bypass; MEND, Multimodal ENhanced Discharge; MVST, multivessel small thoracotomy; SVST, single-vessel small thoracotomy.

Data are reported as median (interquartile range) or *n* (%).

**Fig. 3.**
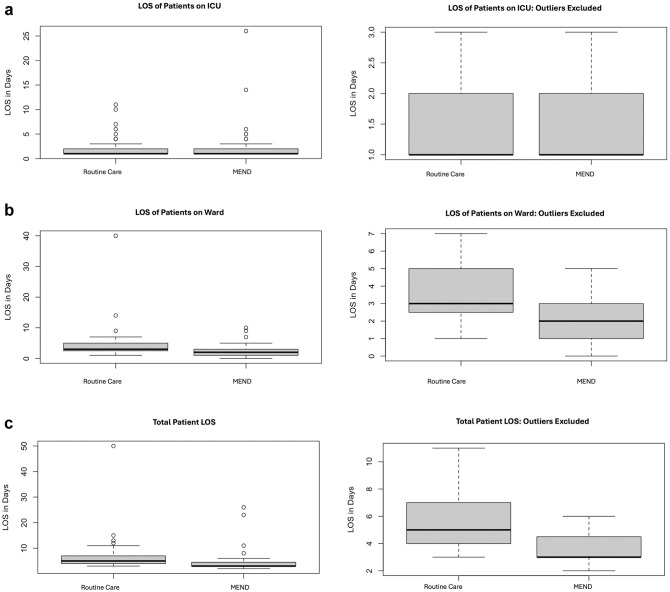
Boxplots illustrate all data points (left) and data with outliers excluded (right) for each LOS category investigated including (a) ICU LOS, (b) ward LOS, and (c) total LOS. ICU, intensive care unit; LOS, length of stay; MEND, Multimodal ENhanced Discharge.

**Fig. 4.**
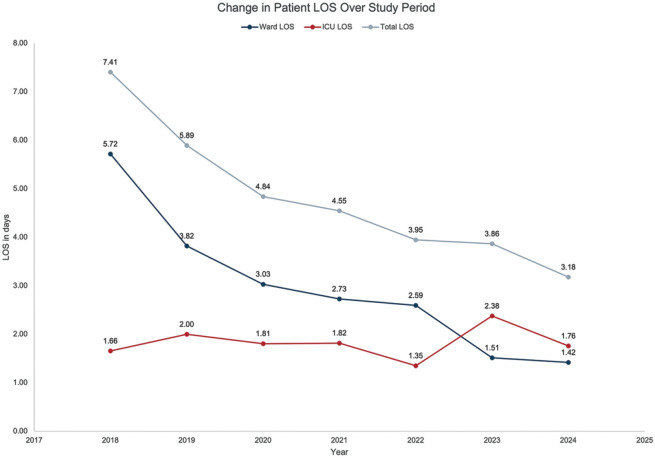
Decrease in mean patient LOS as a function of time from 2018 to 2023. ICU, intensive care unit; LOS, length of stay.

The LOS, reported as medians as well as means ± standard deviation, is captured in Table 3 and illustrated in Figure 3 and Figure 4. The median ICU LOS was 1 day in both cohorts. Median ward LOS was 33% shorter in the MEND group (2 days) versus 3 days in the RC group, which was statistically significant (*P* < 0.001). The median hospital LOS for RC patients was 5 days in comparison with 3 days for MEND patients, which was a statistically significant 40% shorter total LOS for MEND patients (*P* < 0.001). There was no significant difference in readmission rates between the 2 groups (14.3% of RC group [*n* = 13] and 6.6% of MEND group [*n* = 7], *P* = 0.12).

**Table 3.** Summary Statistics of LOS for Routine Care and MEND Patients.

	Routine care *n* = 91	MEND *n* = 107	*P* value
Median LOS, days			
ICU	1 (1–2)	1 (1–2)	0.85
Ward	3 (2.5–5)	2 (1–3)	<0.001
Total	5 (4–7)	3 (3–4.5)	<0.001
Mean LOS, days			
ICU	1.8 ± 1.8	1.9 ± 2.8	0.91
Ward	4.2 ± 4.2	2.3 ± 1.6	<0.001
Total	6.1 ± 5.1	4.1 ± 3.2	0.002
Total readmissions	13 (14.3)	7 (6.5)	0.12

Abbreviations: ICU, intensive care unit; LOS, length of stay; MEND, Multimodal ENhanced Discharge.

Data are reported as median (interquartile range), mean ± standard deviation, or *n* (%).

## Discussion

Patients with CAD undergoing CABG often present with multiple coexisting health conditions and are typically elderly. The physiological and psychological stress induced by surgery is further magnified in cases of frailty or increased complexity. By avoiding sternotomy, MICS CABG has already been shown to reduce surgical wound complications, lower transfusion rates, and accelerate recovery.^4,6,10^ In this study, we report a further significant reduction in hospital LOS without greater readmission rates with the implementation of the dedicated MEND program for MICS CABG patients in comparison with RC.

General postoperative management after CABG involves several key objectives: maintaining stable hemodynamics, controlling heart rate and rhythm, weaning from supplemental oxygen, achieving adequate analgesia, restoring mobility, meeting nutritional goals, ensuring healing surgical wounds, and implementing secondary prevention measures for CAD.^14^ Realizing a low hospital LOS after MICS CABG, despite the nonsternotomy approach, requires a targeted, comprehensive perioperative strategy.^32^

In our institution, the goal of reducing LOS after MICS CABG had been identified in the years prior to implementing MEND, and modest decreases in LOS were observed. This provided the foundation for a formalized perioperative care plan culminating in the development of MEND. The MEND program is rooted in ERAS pathways that promote active recovery and expedited discharge.^15,30^ However, MEND extends beyond ERAS by ensuring that a single, MICS CABG team follows patients through all phases of care, aligning with the CCM. Research has shown that maintaining a longitudinal relationship between patients and a consistent clinician or care team improves quality, intensifies preventative care, and reduces avoidable hospitalization and emergency visits.^21,33^ Another defining feature of the MEND program is its early initiation, at the time of the surgical referral, maximizing the timeline to target patient partnership, optimization, and redirection of care to consultants while respecting the individual patient context. This approach aligns with the MDM model, which advocates for “the adjustment of protocols and practice guidelines to fit evidence-based patient needs, informed patient wants, and complicated patient circumstances.”^17^

As shown in Figure 4, the implementation of MEND led to markedly shorter LOS, without an increase in readmissions. The most significant contributor to this decrease in LOS was the reduction in the duration of stay on the ward. While ICU stay remained similar in both groups studied, we believe that extending MEND into the ICU, resulting in discharge to home from the ICU, played a role in the overall shorter LOS that was observed. An unintended but positive consequence of being able to discharge MEND patients home directly from the ICU was that during periods of limited ward bed availability, other surgical patients who required a longer ward stay were prioritized for transfer out of the ICU.

Beyond enabling enhanced recovery, MEND shaped patient expectations and timelines for recovery after surgery. Formalized in-person follow-up at 1 week after discharge likely mitigated anxieties related to a short hospital LOS after surgery and may have reduced readmissions. Patients in the MEND group had a 6.6% readmission rate compared with 14.3% in the RC group. The sustained decrease in LOS and readmission rates over time—particularly with a relatively small cohort—suggests a causal relationship and highlights the program’s sustainability. Future research tracking patient perceptions and satisfaction could provide valuable insight into the MEND program’s role in reducing readmissions.

It was essential to establish a culture that embraced a short LOS as the norm for both patients and the care team in order to reap the benefits of improved throughput while preserving patient satisfaction.^34^ A major consideration in the development of MEND was to avoid delaying surgery, which could result in hesitancy among both clinicians and patients for program uptake. In the years observed, the median wait time between cardiac catheterization and surgery was statistically similar between the RC (45.0 [IQR 13.0 to 93.5] days) and the MEND (42.5 [IQR 10.0 to 85.0] days) group (*P* = 0.69), confirming that MEND did not prolong the timeline for surgery.

In our hospital, the cost per night stay in the ICU is $8,007, and on the ward is $2,163. Overall, the roughly 2-day reduction in the LOS has significant implications for improving organizational flow and reducing systemic costs; however, a more robust cost analysis should be conducted in future studies.

One limitation of this study is the potential for expertise bias as the surgeon’s experience may have evolved over the 6-year study period. However, because the series was preceded by more than 400 MICS CABG cases in which LOS had not appreciably changed, the standard of surgical performance before and after the implementation of MEND was assumed to be comparable.

Although the impact of the COVID-19 pandemic may be a statistically confounding variable due to mandated capacity reductions during lockdown periods, its onset spurred strong support for enhancing hospital efficiencies to reduce potential COVID-19 exposure by reducing patient stays for elective MICS CABG.

Given that the MEND program has emerged as the standard of care for MICS CABG patients at our hospital, conducting a prospective study to assess its impact would be impractical. However, future research could include propensity-matched analyses and multicenter randomized controlled trials, using both retrospective and prospective methodologies in institutions where MEND has not yet been implemented.

## Conclusions

Developed using the ERAS framework and integrating the CCM and MDM principles, neither of which are components of ERAS, the MEND program distinguishes itself as a unique perioperative care model that effectively reduced LOS without increasing readmission rates in our MICS CABG program. MEND is a scalable and generalizable program with the potential to enhance patient satisfaction, expand institutional capacity, and reduce cost and surgical wait times. Although originally designed for MICS CABG, its strategies for reducing LOS and readmissions could successfully be adapted to sternotomy-based surgical revascularization programs.
